# A scoping review: Understanding brain tumor patients’ decisional needs and preferences

**DOI:** 10.1093/nop/npaf056

**Published:** 2025-06-17

**Authors:** Iris J M Bras, Petra Hoogendoorn, Margriet M Sitskoorn, Geert-Jan M Rutten, Karin Gehring

**Affiliations:** Department of Neurosurgery, Elisabeth-TweeSteden Hospital, Hilvarenbeekse Weg 60, 5022 GC Tilburg, the Netherlands; Department of Cognitive Neuropsychology, Tilburg School of Social and Behavioral Sciences, Tilburg University, Warandelaan 2, 5000 LE Tilburg, the Netherlands; National eHealth Living Lab, Department of Public Health and Primary Care, Leiden University Medical Center, Albinusdreef 2, 2333 ZA Leiden, the Netherlands; Department of Cognitive Neuropsychology, Tilburg School of Social and Behavioral Sciences, Tilburg University, Warandelaan 2, 5000 LE Tilburg, the Netherlands; Department of Neurosurgery, Elisabeth-TweeSteden Hospital, Hilvarenbeekse Weg 60, 5022 GC Tilburg, the Netherlands; Department of Neurosurgery, Elisabeth-TweeSteden Hospital, Hilvarenbeekse Weg 60, 5022 GC Tilburg, the Netherlands; Department of Cognitive Neuropsychology, Tilburg School of Social and Behavioral Sciences, Tilburg University, Warandelaan 2, 5000 LE Tilburg, the Netherlands

**Keywords:** brain tumor patients, decision aids, decisional needs, decisional preferences, scoping review

## Abstract

**Background:**

Informed, value-based healthcare decisions depend on individuals’ decisional needs and preferences. Adequately addressing them may improve decision experience and quality. This scoping review aims to assess study findings on decisional needs and preferences of patients with a brain tumor (PwBT) (glioma, meningioma, brain metastases) throughout the disease trajectory, including findings on decision aids and interventions.

**Method:**

The methodological framework by Arksey & O-Malley for scoping reviews was used. A systematic search was performed in PubMed.

**Results:**

We identified 20 studies on decisional needs and preferences of PwBT and 6 studies on decision aids. Most patients prefer a collaborative or active role in decision-making, and they value quality of life (QoL), functional independence, and survival as treatment outcomes. Patients require tailored amounts and types of information and need support maintaining hope, establishing trust, and with diminished medical decision-making abilities. Decision aids focused on information provision or shared decision-making (SDM), with mixed results on patient participation and satisfaction.

**Discussion:**

SDM could help address PwBT’s needs and preferences. QoL and functional independence are crucial yet underexplored factors in decision-making. Further research is needed to better integrate individual patient outcome preferences into SDM and to evaluate tools that support informed and value-based decisions.

**Key Points**

1. Most brain tumor patients wish to be involved in decision-making.
2. Patients need tailored information and support from healthcare professionals and family caregivers.
3. Decision aids focused on improving information provision or shared decision-making.

Decision-making in brain tumor treatment is complex and requires a comprehensive understanding of patients’ needs and preferences. First, treatment options are limited and often noncurative.^1^ The available treatments are aimed at stabilizing and controlling the tumor, such as surgery, radiation, and chemotherapy, and will on average prolong life to some extent but may also exacerbate symptoms and reduce quality of life (QoL).^2,3^ Therefore, the viability, benefits, and risks of all options, including no active tumor-directed treatment focused on symptom management and optimizing QoL, or a wait-and-scan approach, should be carefully weighed against one another. Second, patients and family caregivers are confronted with a serious illness that may substantially reduce life expectancy and/or disrupt daily family and working life, often resulting in high levels of psychological distress surrounding decision-making.^4,5^ Lastly, the brain tumor and its treatments may impair patients’ cognitive functioning.^4,6^ Impaired cognitive functioning can result in diminished decision-making capacity, referring to a person’s ability to understand relevant information, assess the consequences of options, and make informed choices about their healthcare.^4,7^

There is growing recognition of the importance of involving patients in decision-making, a concept known as shared decision-making (SDM).^2,8,9^ With SDM, bi-directional information exchange takes place: patients share preferences for decision-making and treatment outcomes that matter to their daily lives, and healthcare professionals (HCPs) discuss what care would be best aligned with the disease and these patient values.^9,10^ Nevertheless, the specific needs and preferences of patients with a brain tumor (PwBT) and their family caregivers in decision-making remain underexplored.^11^ Decisional needs and preferences refer to specific requirements individuals have to make informed, value-based healthcare decisions, as outlined in detail in the Ottawa Decision Support Framework (ODSF) and guide developed for use by practitioners and patients.^12,13^ In summary, these needs include access to decision-specific information about available options, their benefits, risks, and potential outcomes. Additionally, they involve clarification of patient outcome preferences to weigh the relative importance of the benefits, risks, and outcomes associated with each option. Furthermore, needs and preferences regarding self-efficacy, defined as confidence in one’s ability to actively participate in the decision-making process, are important for patients to engage meaningfully in treatment decision-making. Finally, support needs are critical, encompassing guidance from HCPs and family caregivers.^13^

Research has shown that adequately addressing decisional needs and preferences may improve decision experience and patient outcomes.^12,14^ Previous reviews on SDM in high-grade glioma patients and neurosurgical patients suggest that satisfactory involvement of PwBT in decision-making can have beneficial effects on patient-centered outcomes,^8^ such as reduced decisional uncertainty and improved social and emotional well-being.^15,16^ Furthermore, a review across various patient populations, including 105 studies on decision aids, reported that patients who used decision aids improved their knowledge of options, felt better informed, and were more certain about what matters most to them. It also appeared that patients had more accurate expectations of the benefits and harms of options, participated more actively in decision-making and that healthcare choices aligned better with patient values.^14^

This scoping review aims to assess study findings on decisional needs and preferences of PwBT throughout the disease trajectory, including findings on decision aids or interventions.

## Methods

### Methodological framework

Scoping reviews are intended to map key concepts that underpin a research area, without extensively assessing its methodological quality or risk of bias. The methodological framework for scoping reviews by Arksey & O-Malley was employed, following the stages outlined in their framework: (1) identifying the broad research question; (2) identifying relevant studies; (3) selecting studies; (4) charting the data; and (5) collating, summarizing, and reporting the results.^17^

### Search strategy

A broad search strategy was developed and refined, since the terminology used for decisional needs, preferences, and decision aids or interventions varies considerably (Appendix 1). Search terms had to be included in the title and/or abstract. A systematic search was performed in PubMed (10th of December 2024).

### Eligibility criteria

Studies were included if they were full-text, published, written in English, and peer-reviewed empirical studies on an adult (>18 years) patient population with a majority of patients with low- or high-grade glioma, meningioma, or brain metastases. Case reports, personal narratives, editorials, commentaries, and reviews were excluded. Studies had to cover the patient perspective on decisional needs and preferences or decision aids and interventions targeting patients. This included studies where family caregivers, advocacy leaders, or HCPs provided the patients’ perspective. Family caregivers can be relatives, friends, or neighbors who provide care.^18^ This scoping review will not evaluate patient-reported outcomes regarding SDM, as they have been thoroughly explored for PwBT in 2 recent reviews.^2,8^ Furthermore, studies solely focusing on late palliative care where treatments were no longer viable for patients, were not included. No restraints were set for the date of publication.

### Selection process

Retrieved records were processed in the software program Endnote (Endnote X9, Clarivate Analytics). Duplicates were removed. The first author (I.B.) reviewed the studies by title and abstract. Potentially eligible studies and studies with a title and abstract that carried insufficient information to determine eligibility were read as full-text articles and assessed against inclusion criteria by the authors I.B. and K.G. In addition, the references of the included articles and other possibly relevant articles, such as reviews with adjacent topics, were hand-searched.^2,8,19^ In case of disagreement regarding inclusion, consensus on which articles to include was reached by discussion between I.B. and K.G. A Preferred Reporting Items for Systematic Reviews and Meta-analysis extension for Scoping Reviews (PRISMA-ScR) flowchart was created.^20,21^

### Data extraction, analysis, and synthesis of results

Data extraction on decisional needs and preferences, decision aids, and interventions was performed, and 2 tables were created: 1 for studies on decisional needs and preferences and 1 for studies on decision aids and interventions. Furthermore, general information on authors, year of publication, country, sample characteristics and size, timing in the disease trajectory, objectives for collecting data, methods used, and main findings concerning the aim of this scoping review were collected. We conducted a narrative synthesis of results, grouping the data into categories of different needs and preferences: Decisional role, Treatment outcomes, Information, Practical and emotional support, and Decision aids and interventions. Within each category, the results were narratively summarized. The review is reported according to the PRISMA-ScR^21^ (Appendix 2).

## Results

**Figure 1.**
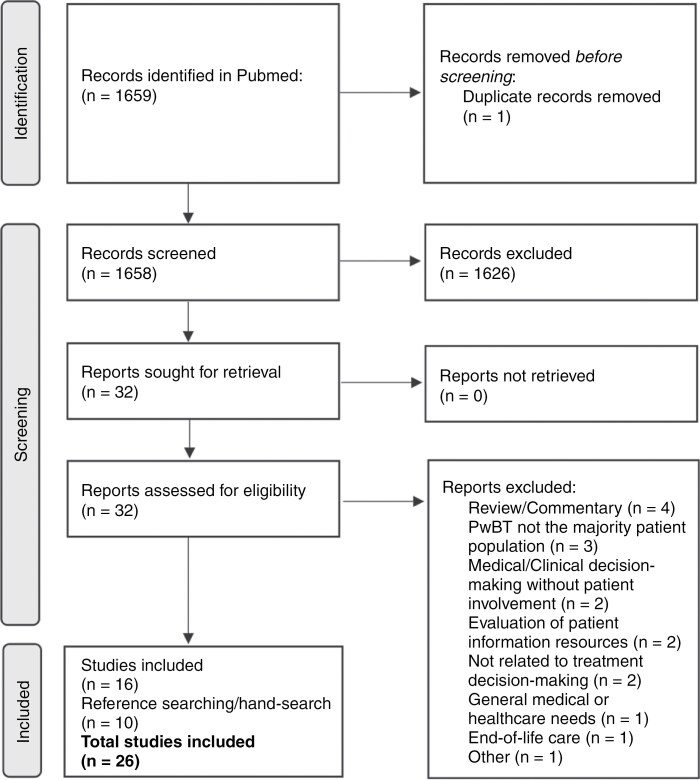
Inclusion process following a systematic search. Abbreviations: PwBT = patients with a brain tumor.

A total of 1659 articles were identified through a PubMed search. A total of 32 articles were assessed in full text for eligibility, of those 16 articles were excluded. Ten additional studies were identified through reference screening and handsearch. See the PRISMA flowchart (Figure 1) for reasons for exclusion and additional information on the selection process.

Fifteen studies included patients with one type of diagnosis, being glioma patients (*n* = 11),^11,22–31^ and brain metastases patients (*n* = 4).^32–35^ No studies focused solely on meningioma patients. Another 11 studies comprised a mixed sample of PwBT (*n* = 11).^36–46^ These samples consisted of patients with different types of brain tumor diagnoses (*n* = 6),^37,40,42,44–46^ in part PwBT and in part other patient populations (*n* = 2),^36,39^ or specific brain tumor diagnoses were not reported (*n* = 3).^38,41,43^ A total of 740 patients with the searched brain tumor diagnoses (glioma, meningioma, brain metastases) were included in all articles, with an additional 321 family caregivers, 7 advocacy leaders, and 5 HCPs providing insight into the patient perspective.

Of these studies, 15 adopted a qualitative approach,^11,22–27,29,31,33,35,38,39,41,44^ 8 reported quantitative results only,^32,34,37,40,42,43,45,46^ and 3 combined qualitative and quantitative approaches.^28,30,36^ Twelve studies used semi-structured interviews,^11,22,23,25,28,30,31,33,35,39,41,43^ 4 studies (repeated) in-depth interviews,^26,29,36,38^ 1 study semi-structured interviews in combination with 2 focus groups,^27^ and 1 study used a roundtable discussion without specifying the set-up details, such as how participants were selected or how the discussion was carried out.^24^ Three studies used the Control Preferences Scale (CPS),^47^ to assess patients’ preferred role in the decision-making process on a 5-point Likert scale ranging from making the decision alone to sharing it with or delegating it to the HCP.^28,36,37^ One study used the CollaboRATE questionnaire to measure the perceived level of SDM by patients,^46^ 1 study used the Preparation for Decision-Making Scale (PDMS),^30^ and 6 studies used self-developed questionnaires.^32,34,40,42,44,45^

Twenty studies provided results on decisional needs and preferences of PwBT (Table 1)^11,22–28,32–43^ and 6 on decision aids and support interventions for PwBT (Table 2).^29–31,44–46^ All included studies employed prospective or cross-sectional designs. Eleven studies focused on newly diagnosed patients within 1 year of diagnosis,^23,29,31–33,35–38,42,46^ such as when deciding on first treatment,^32,36,37,46^ when patients were hospitalized for the first time for brain surgery,^38^ surrounding the first neurosurgical intervention^31,42^ or consultations following initial treatments.^29^ One study focused explicitly on patients with recurrent disease,^11^ and 12 studies included patients at various stages of the disease trajectory,^22,25–28,30,34,39–41,44,45^ though only 1 study provided the mean elapsed time since diagnosis.^27^ One study did not report any information on the timing of the disease trajectory.^43^

**Table 1.** Studies on decisional needs and preferences of patients with a brain tumor.

Author, year reference (country)	Sample size of pts with diagnoses, family caregivers, or HCPs	Timing in the disease trajectory	Aim of the study (relevant to the scoping review)	Method	Findings (relevant to the scoping review)
Boele et al., 2023^22^ (United Kingdom)	*n* = 15 Pts with GBM *n* = 13 Family caregivers *n* = 5 HCPs Results presented on whole group	Various stages from <1 year following diagnosis to >2 years	To explore experiences and preferences around GBM treatment communication	Semi-structured interviews ^a^	**Decisional role** Follow HCPs advice. Be involved in DM and voice what matters to them and their families. Chemo- and/or radiotherapy were often interpreted as the only option. To have a say in deciding to stop or pause treatment. **Support** Risks were overemphasized and not balanced against potential benefits. To maintain some sense of control. Form a trusting relationship with the treatment team. **Information** To understand treatment procedures to plan life around it and to know whether treatment is the best or the only option. **Treatment outcomes** Valued extending life when QoL could be preserved. Reconciling realistic treatment outcomes and maintaining hope was difficult.
Brom et al., 2014^36^ (Netherlands)	*n* = 18 Pts with high-grade glioma *n* = 10 Pts with metastatic colorectal cancer Results presented on whole group	Starting with first line treatment of chemotherapy	To gain insight into cancer pts preferences and the reasons for pts preferred role in treatment decision when discussing chemotherapy to prolong survival, control symptoms, and/or maintain or improve QoL	CPS^b^ In-depth interviewing^a^	**Decisional role** No pts wanted to make the decision completely independent of their HCP. Active role, 32.1% (*n* = 9): Pts wanted to be in control over their own life or felt that they could not defer this responsibility to their HCP. Collaborative role, 35.7% (*n* = 10): Pts placed more emphasis on reaching a consensus with the physician, emphasizing their own importance in the process as experts on who they are. Passive role, 28.6 % (*n* = 8): Pts emphasized the importance of the expertise of their physician and their own limited medical knowledge. No preference indicated, 3.6% (*n* = 1). Most pts thought it was difficult to indicate their preferred role as it dependent on the situation and the type of decision. Pts predicted that they would adopt a more active role in DM in the later phases of their disease when options for life-prolongation would become limited, and QoL would take precedence.
Cavers et al., 2013^26^ (United Kingdom)	*n* = 26 Pts with high- and low-grade glioma *n* = 23 Family caregivers Results presented on whole group	From before histological diagnosis to bereavement	To describe insights into pts’ and their families’ emotional experience of illness, information, and support needs	Serial interviews conducted over one year (1–5 interviews) ^a^	**Support** Ability to absorb information was often affected, especially in early phase when shock and a cognitive “shutdown” inhibited information absorption and retention. Strategic in handling of information and sought only positive information to create a sense of hope. Supportive relationships and communication enabling pts to maintain hope over time were critical, especially in the early phase. HCPs’ manner of delivering information was central to maintaining hope, and participants did not always feel it was handled sensitively. Perceived lack of reassurance and emotional support, instead focusing on physical care was distressing. **Information** Experienced conflict between wanting information and not ready to take in difficult information.
Dorman et al., 2009^35^ (United Kingdom)	*n* = 9 Pts with nonsmall cell lung cancer with brain metastases where oncologists were uncertain about the benefit of WBRT	Recent radiological diagnosis of brain metastases (within 4 weeks prior)	What pts want from their treatment	Semi-structured interviews^a^	**Treatment outcomes** Quality vs quantity of life: -As good as possible for as long as possible -As long as possible whatever the quality - Living life to the full then rapid deteroriation—avoiding prolonged dying phase Factors important to QoL: family, mobility/movement, body image or self-image, cognitive function, freedom, normality, and ability to work. Fear of loss of dignity, dependence, being a burden, being pitied or being in pain.
Finocchiaro et al., 2011^43^ (Italy)	*n* = 18 PwBT No further specifications regarding the diagnoses were provided *n* = 17 Family caregivers	Not reported	To study effective knowledge of diagnosis and treatment	Semi-structured interviews^b^	**Information** 68.4% wanted to be informed about their condition. 15.5% would rather not be informed, but have the information provided to a relative. 5.9% wished to be the only person informed about their condition.
Goebel and Mehdorn, 2018^42^ (Germany)	*n* = 42 Mixed sample of PwBT: *n* = 12 GBM *n* = 13 Meningioma *n* = 4 Lymphoma *n* = 13 Other (Oligodendroglioma, Schwannoma)	After the first neurosurgical treatment	To assess pts’ preferences for the communication of bad news	Self-developed questionnaire^b^	**Support** Pts reported 39.7 communication preferences on average (SD: 6.65, range 13–46). 27 items of the questionnaire were reported by >90% as a preference. The three most important ones were “being told in person,” “doctor giving me full attention,” “doctor maintaining eye contact,” all reflecting pts’ need for emotional support.
Hack et al., 2021^37^ (Canada)	*n* = 133 Mixed sample of PwBT: *n* = 70 GBM *n* = 10 Astrocytoma *n* = 9 Oligodendroglioma *n* = 9 Meningioma *n* = 16 Other diagnoses	First treatment consultation after diagnosis	To check for decisional control preference differences between 2 groups testing an intervention	CPS^b^	**Decisional role** Active role: 39.8% (*n* = 53) Collaborative role: 45.9% (*n* = 61) Passive role: 14.3% (*n* = 19) No difference between groups.
Halkett et al., 2010^23^ (Australia)	*n* = 19 Mixed sample of PwBT: *n* = 16 GBM *n* = 1 Astrocytoma *n* = 2 Anaplastic gemistocytic astrocytoma	<1 year of diagnosis, undergoing chemo-radiotherapy	To understand pts’ experiences with high-grade glioma and identify and describe information and support needs	Semi-structured interviews^a^	**Support** Characterized by communication deficits. Reliance on family caregivers. **Information** Uncertainty about prognosis. Appreciated the opportunity to gain information and ask questions. **Treatment outcomes** Concerned about seizures, vision loss, memory loss, speech difficulties, mobility, return to work, financial stability, and resumption of activities.
Kumthekar et al., 2024^28^ (United States)	*n* = 40 GBM	*n* = 32 Utilized TTFields, *n* = 8 Not utilized TTFields	To examine pt and clinician views of TTFields and factors shaping utilization of TTFields	CPS^b^ Semi-structured interviews by telephone^a^	**Decisional role** Pt makes decision (active role): 7.5% (*n* = 3) Pt makes decision after considering HCPs opinion (active role): 50% (*n* = 20) Pt and HCP make decision (collaborative role): 27.5% (*n* = 11) HCP makes decision but considers pts opinion (passive role): 7.5% (*n* = 3) HCP makes decision (passive role): 7.5% (*n* = 3) **Treatment outcomes** Top 3 reasons for using TTFields Efficacy: 71.9% (*n* = 23) Doctor’s opinion: 28.1% (*n* = 9) Family considerations: 12.5% (*n* = 4) Top 3 reasons for declining TTFields Shaving head: 50% (*n* = 4) Visibility: 37.5% (*n* = 3) Inconvenience of carrying device and concern of interaction with other treatment: both 25% (*n* = 2)
Lepola et al., 2001^38^ (Finland)	*n* = 8 PwBT Diagnosis not known at the time of the interview	First time being admitted to the hospital for brain surgery	To describe the experience of being a pt in the neurosurgery clinic	Repeated interviews, the day before and 3–7 days after surgery, two per person^a^	**Decisional role** Varied desire to participate in DM among pts. Some wanted to delegate this to medical experts. To be provided with alternative options and to offer input about pain relief, meal arrangements, choice of room, and radiotherapy.
Musella et al., 2021^24^ (United States)	*n* = 7 PwBT advocates that provided support to PwBT from the following organizations: Musella Foundation for Brain Tumor Research & Information, the National Brain Tumor Society, the American Brain Tumor Association, CancerCare, Head for the Cure Foundation, and the EndBrainCancer Initiative	N/A	To discuss how to effectively implement SDM into practice for GBM pts.	Roundtable discussion. No details on set-up reported^a^	**Decisional role** SDM should start with initial encounter with neurosurgeon. **Support** Diminished cognitive abilities or emotional response to disease may impact DM abilities. **Treatment outcomes** Care does not always reflect pt goals, ie pt preferences for level of aggressive treatment and/or important personal goals or milestones.
Papadakos et al., 2019^34^ (Canada)	*n* = 124 Pts with brain metastases *n* = 81 Family caregivers Results presented on whole group	From newly diagnosed to recently completed treatment for recurrence.	To assess informational care needs	Self-developed questionnaire regarding pt informational and supportive care needs [range score 0–100]^b^	**Information** Pts prioritized medical (mean 67.1, eg information on medical follow-up and treatment options) and physical (66.5, eg information on location of the tumor and symptom management) information, followed by practical (42.6) and emotional (40.3) information, while social (30.8) and spiritual (28.1) domains were rated lowest.
Reinert et al., 2018^40^ (Germany)	*n* = 172 Mixed sample of PwBT: *n* = 34 GBM *n* = 48 Astrocytoma *n* = 31 Oligoastrocytoma / Oligodendroglioma *n* = 19 Meningioma *n* = 40 Other *n* = 142 Family caregivers Results presented on whole group	Pts currently under treatment were approached within a set period of 1 year (regardless of the date of their first diagnosis)	To evaluate information needs	Self-developed questionnaire^b^	**Information** 59.9% wished to be fully informed. 18.6% rather wished a comprehensive explanation of diagnosis and treatment. Pts want to be informed on current treatment options, alternative and innovative treatment approaches, and alternatives to chemotherapy but also on supportive treatments or side effects of treatment.
Rozmovits et al., 2010^39^ (Canada)	*n* = 25 Pts with nonmalignant, but life-threatening, intracranial lesions: *n* = 10 Meningioma *n* = 7 Vestibular schwannoma *n* = 4 Aneurysm *n* = 3 Arteriovenous Malformation *n* = 1 Colloid Cyst	2–24 months after brain surgery	To explore information needs and to provide evidence to support the development of more patient-centered approaches to doctor-patient communication by neurosurgeons	Semi-structured interviews ^a^	**Decisional role** Range between wanting to be actively involved to leaving the decision to the neurosurgeon. **Support** Compassion from their neurosurgeon. **Information** More detailed and more voluminous information needed. Want information about treatment options, surgical risks, neurosurgeon’s background, reputation, experience, an overview of the actual surgical procedure and expectations for their recovery, both short and long term.
Succop Jr. et al., 2023^27^ (United States)	*n* = 20 Pts with glioma: *n* = 6 Glioma grade 1 *n* = 5 Glioma grade 2 *n* = 4 Glioma grade 3 *n* = 5 Glioma grade 4 *n* = 3 Family caregivers Results presented on whole group	Mean elapsed time since diagnosis 45 ± 29 months	To understand how pts value cognitive treatment outcomes.	2 focus groups, three dyad interviews and eleven individual interviews^a^	**Support** Reliance on loved ones and support groups when deciding on pursuing treatment. **Treatment outcomes** Losing all independence was a treatment dealbreaker.
Sjövall et al., 2024^41^ (Sweden)	*n* = 22 PwBT No further specifications regarding the diagnoses were provided	Received proton beam therapy	To explore pts’ experiences of participation in the treatment decision of proton beam therapy vs conventional radiotherapy	Semi-structured interviews^a^	**Decisional role** Theme: to be a voice that matters. Theme: to get control over what will happen. Theme: feeling of not having medical expertise and therefore handing over the decision to doctors.
Sørensen von Essen et al., 2022^11^ (Denmark)	*n* = 29 Pts with high-grade glioma: *n* = 15 GBM *n* = 2 Anaplastic astrocytoma *n* = 3 Anaplastic oligodendroglioma *n* = 14 Family caregivers Results presented on whole group	Recurrent disease	To explore the decisional needs to guide clinicians in providing future decision support and SDM	Semi-structured interviews^a^	**Decisional role** To be involved in DM regarding treatment and care. DM responsibility was experienced as a burden. Need to rely on the surgeon’s expertise and delegated DM responsibility to the neurosurgeon. Acknowledged that decision could have impact on family life, but that the pt has the final word in making the decision. **Support** Family played a significant role practically and emotionally. Especially for pt with aphasia or other cognitive impairments. Pts emphasized that clinicians should never take away a patient’s hope. Having a trustful relationship with the neurosurgeon could directly influence the participants’ decision about surgery. **Information** Balance of information and the words used to describe the tumor or surgical procedure affected participants’ risk assessment and treatment decision. Participants stressed that receiving adequate information delivered in an understandable way was vital. Preferences regarding the type and amount of information differed. Important to individually tailor information. **Treatment outcomes** Participants valued QoL over prolonging life, but this was not necessarily reflected in their treatment decisions. The hope of prolonging life affected their willingness to accept or reject the potential treatment risks.
Sterckx et al., 2015^25^ (Belgium)	*n* = 17 Pts with high-grade glioma No further specifications regarding the diagnosis were provided	*n* = 8 First-line treatment *n* = 8 Second-line treatment after relapse or of progressive disease *n* = 1 No treatment	How do pts experience living with HGG and what are their needs related to professional care?	Semi-structured interviews^a^	**Decisional role** Felt excluded from decisions concerning their care. Feelings of disregard occurred when family caregivers or HCPs failed to involve pts in decision or when family caregivers were preventing them from being well-informed.
Sze et al., 2006^33^ (Canada)	*n* = 20 Pts with brain metastases *n* = 19 Family caregivers Results presented on whole group	Within the first two months of diagnosis	To describe major factors important in DM for WBRT for pts and family caregivers at the time of radiation consultations	Semi-structured interviews (one)^a^	**Support** The nature of hope varies depending on the pt, hope for a cure, increased life expectancy, improved symptom management and acceptance. **Information** Information preferences between pts varied.
Zeng et al., 2017^32^ (Canada, United States)	*n* = 23 Pts with brain metastases	Deciding on stereotactic brain surgery and stereotactic brain surgery plus WBRT	The goal was to determine the proportion of pts who wish to partake in treatment decisions and their important considerations when making such decisions	Choosing between active/passive role^b^ Self-developed questionnaire concerning treatment considerations, importance ranked from 0 to 10 and an open-ended question^a^	**Decisional role** All pts wished to take an active role in DM. **Treatment outcomes** Factors influencing treatment decisions: (1)QoL (9.4/10) (2)Maintaining functional independence (9.3/10) (3)Survival (9.2/10) (4)Cognition (9.0/10) (5)Side effects (8.4/10) (6)Prevent existing metastases from growing (8.1/10) (7)Prevent existing and new metastases from growing (7.3/10) (8)Risk of developing new metastases (7.3/10) (9)Prevent new metastases from growing (7.0/10) (10)Multiple trips to the hospital (5.0/10)

a Qualitative outcomes.

b Quantitative outcomes. Abbreviations: Pts = patients. HCP = Healthcare professional. GBM = glioblastoma. DM = decision-making. QoL = Quality of life. CPS = Control Preferences Scale. PwBT = patients with a brain tumor. TTFields = Tumor Treating Fields. SDM = Shared decision-making. WBRT = whole-brain radiotherapy.

**Table 2.** studies on decision aids and support for patients with a brain tumor.

Author, year, reference (country)	Sample size of pts with diagnoses, family caregivers, or HCPs	Timing in the disease trajectory	Aim of the study (relevant to scoping review)	Method	Findings (relevant to the scoping review)
Bergers et al., 2024^44^ (Netherlands)	*n* = 42 Mixed sample of PwBT: *n* = 19 GBM *n* = 8 Meningioma *n* = 7 Glioma *n* = 5 Brain metastases *n* = 2 Other *n* = 1 Unknown	*n* = 8 experienced recurrence of disease Timing of self-developed questionnaire administration unclear: ranges from before first consultation to during active treatment	Evaluation of usefulness of the person-centered care tool: “we would like to know you”	Content analysis of self-developed questionnaire: 5 open questions ^a^	Frequently mentioned topics for important activities: social activities, sports, or maintaining normal activities. Important people: partner, children, relatives, or friends. Concerns: influence of disease on physical and mental health or disrupting daily life activities. Fear of the future in general. Wanted HCP to know healthcare preferences, such as treatment goals or communication preferences.
Hertzsprung et al., 2023^45^ (Germany)	*n* = 27 Mixed sample of PwBT: *n* = 9 Meningioma *n* = 8 Brain metastases *n* = 6 High-grade glioma *n* = 4 Pituitary adenoma	Pts scheduled for a resection regardless of prior treatment No further specifications provided	To investigate potential benefits of additional stereoscopic visualization of patient-specific imaging during surgical informed consent conversation prior to a resection	Additional 3D stereoscopic visualization of patient-specific imaging Self-developed questionnaire using 5-point Likert scales on experience and objective knowledge^b^	No statistically significant differences were found for improved experiences or objective knowledge about the surgical procedure.
Leu et al., 2023^46^ (United Kingdom)	*n* = 96 Mixed sample of PwBT: *n* = 49 High-grade glioma *n* = 10 Low-grade glioma *n* = 24 Brain metastases *n* = 13 Missing histology	First neurosurgical consultation	To analyze satisfaction with the SDM process for brain tumor surgery before and after a SDM training with tools	Decision-grids including 3 different treatment options CollaboRATE questionnaire^b^	Nonsignificant change in mean CollaboRATE score before 25.82/27 [range 15–27] after 26.27/27 [range 11–27] (*P* = .23)
Shepherd et al., 2023^29^ (United Kingdom)	*n* = 12 Pts with high-grade glioma: *n* = 10 GBM *n* = 2 Anaplastic oligodendroglioma	Initial diagnosis consultation following surgery, 3-month follow-up appointment following surgery and consultation following chemotherapy treatment (for participants that had chemotherapy)	To assess the impact of CPRS approach.	CPRS Three semi-structured interviews^a^	Consultation planning supported pts to generate important questions and information to share with their HCPs. This helped them receive meaningful information from their HCP and united pts and significant others to ensure a shared agenda. Provision of consultation summary and recording reassured participants. It enabled paced information uptake. Although supported in readiness for SDM, they did not feel they had a meaningful choice to make..
Sørensen von Essen et al., 2022^30^ (Denmark)	*n* = 11 Pts with high-grade glioma: *n* = 10 GBM *n* = 1 Anaplastic astrocytoma *n* = 9 Family caregivers *n* = 11 HPCs	Primary treatment or follow-up, *n* = 5 Treatment or follow-up after recurrence, *n* = 6	To develop a decision aid to support SDM	Alpha-test by potential users Structured interviews^a^ PDMS questionnaire (9 items)^b^	Participants in alpha test were generally positive towards the decision aid and implementation in clinical practice. PDMS score pts and family caregivers [0–100]: 80.83. Assessed decision aid to have a high level of perceived preparation for DM.
Van de Belt et al., 2018^31^ (Netherlands)	*n* = 11 Pts with glioma: *n* = 8 Glioma grade 2 *n* = 2 Glioma grade 3 *n* = 1 Unknown	Pts undergoing surgery. No further specifications provided.	To assess what pts value or fear about patient-specific three-dimensional (3D) models of tumors when they are used to educate them about the relationship between their tumor and specific brain parts, surgical procedure, and risks.	Semi-structured interviews^a^	Experiences were predominantly positive, particularly regarding understanding their medical situation, psychological aspects, and communication.

a Qualitative outcomes.

b Quantitative outcomes. Abbreviations: Pts = patients. HCP = healthcare professional. PwBT = patients with a brain tumor. GBM = glioblastoma. DM = decision-making. DCS = Decisional Conflict Scale. SDM = Shared decision-making. CPRS = Consultation Planning, Recording, and Summarizing. PDMS = Preparation for Decision-Making Scale.

### Decisional role preferences

Nine studies provided insight into the role patients wish to adopt in the decision-making process.^11,22,25,32,36–39^ Zeng and colleagues conducted a study involving 23 patients with brain metastases choosing between stereotactic brain surgery alone or combined with whole-brain radiotherapy. When asked about their preference for an active or passive role in the decision-making process, all patients chose an active role.^32^ The 3 studies that employed the CPS depict varying decisional role preferences for a mixed PwBT sample (*n* = 133),^37^ a sample with GBM patients only (*n* = 40),^28^ and a sample including both metastatic colorectal cancer patients (*n* = 10) and high-grade glioma patients (*n* = 18)^36^ ranging from a passive role, where the HCP decides whilst considering the patient’s opinion, (14.3%–28.6%), to a collaborative role, where patient and HCP decide together (27.5%–45.9%) or an active role, where the patient decides alone or whilst considering the HCP’s opinion (32.1%–57.5%).

Moreover, Brom, and colleagues,^36^ in a sample including both metastatic colorectal cancer (*n* = 10) and high-grade glioma patients (*n* = 18), used the CPS as the starting point of in-depth interviews. The aim was to gain insight into the reasons for their preferred role in treatment decision-making when discussing chemotherapy to prolong survival, control symptoms, or optimize QoL.^36^ Most patients of this mixed sample wished to be involved and preferred a collaborative role, although in the interviews it became clear that patients find it hard to choose their role preference as it is highly dependent on the situation and type of decision.^36^ In the study by Boele and colleagues on high-grade glioma patients (*n* = 15), patients reported in semi-structured interviews feeling like they had little choice when deciding on surgery or chemo- and radiotherapy, as these were the only available *treatment* options. They stressed that having a say in when to stop or pause treatment was very important.^22^ A similar notion was put forward by patients with newly diagnosed brain metastases (*n* = 20) and their family caregivers (*n* = 19) in the interview study by Sze and colleagues,^33^ where radiotherapy was seen as the only option available to them and supportive care was not considered a serious alternative.

Variability in the decisional roles that PwBT prefer to adopt is highlighted in other qualitative studies.^11,22,38,39^ On the one hand, results showed that patients wanted and needed to rely on the expertise and advice of the HCP,^11,22,41^ that they wanted to delegate the treatment decision to medical experts,^38,39^ and that decision responsibility was experienced as a burden.^11^ On the other hand, it also became clear that patients wanted to be involved in the decision-making process; for instance, they want to voice what matters to them or their families^22,41^ or wish to know everything^39^ to get a sense of control over the situation.^41^ In the study by Lepola and colleagues,^38^ PwBT without reported diagnosis (*n* = 8) hospitalized for the first time for brain surgery wanted HCPs to give them alternatives to choose from and to provide input about medication, undergoing radiotherapy, meal arrangements, and room choice. Sørensen von Essen and colleagues^11^ found that patients with recurrent high-grade gliomas (*n* = 15) and family caregivers (*n* = 14) agreed in semi-structured interviews that treatment decisions could substantially impact daily life for both of them, yet they also agreed the patient should make the final decision.

The differences in preferred roles could be attributed to how patients perceive their own and their HCP’s responsibility and expertise relevant for decision-making, as explored with semi-structured interviews in the abovementioned study by Brom and colleagues.^36^ High-grade glioma patients (*n* = 18) who preferred an active role felt the need to be in control of their own lives or could not defer this responsibility to their HCP. Whereas high-grade patients selecting a collaborative role emphasized their own expertise about who they are, and patients who selected a passive role emphasized the expertise of the HCP. Additionally, patients predicted that they would adopt a more active role in the later stages of the disease when options for life prolongation would become limited, and QoL would take precedence.^36^ In the study by Musella and colleagues, advocacy leaders (*n* = 7 advocacy organizations) in a roundtable discussion stressed that involving patients in the decision-making process should begin with the initial encounter with the neurosurgeon, as neurosurgical decisions will influence the course of the entire treatment trajectory.^24^

### Treatment outcome preferences

Several studies explored treatment outcomes valued by glioma patients. Boele and colleagues^22^ found through semi-structured interviews that high-grade glioma patients (*n* = 15), family caregivers (*n* = 13), and HCPs (*n* = 5) valued prolonging life when QoL could be preserved. Sørensen von Essen and colleagues^11^ reported that some patients with recurrent high-grade glioma (*n* = 15) even valued QoL over prolonging life, although this preference was not necessarily reflected in treatment decisions, according to patients and family caregivers.^11^ Instead, the hope of extending life led patients to accept potential risks associated with treatment, even when aware of the possible negative impact on QoL. In the study by Musella and colleagues with advocacy leaders on implementing SDM in clinical practice, it was emphasized that care was not always aligned with patient preferences regarding the aggressiveness of treatments, their personal goals, or milestones in life.^24^ In the qualitative study by Halkett and colleagues,^23^ using semi-structured interviews to understand information and support needs, high-grade glioma patients (*n* = 19) undergoing adjuvant treatment were particularly concerned with treatment outcomes regarding seizures, vision loss, memory loss, speech difficulties, and lack of mobility. Additionally, they also referred to their ability to return to work, financial stability, and resumption of previous activities.^23^ Succop Jr. et al.^27^ conducted a study with 20 glioma patients (grades 1–4) to identify cognitive functions most affected by treatment and those most important to patients’ QoL. The study, using 2 focus groups, 3 dyad interviews, and 11 individual interviews, found that a key treatment dealbreaker for patients was the prospect of losing all independence. Kumthekar et al.^28^ conducted semi-structured telephone interviews to examine patient views on whether to opt for Tumor Treating Fields (TTFields), a cancer treatment using low-intensity electric fields to disrupt cancer cell division in GBM patients (*n* = 40). Among those who used TTFields (*n* = 32), the main reasons for usage were efficacy (71.9%), the doctor’s opinion (28.1%), and family considerations (12.5%). The primary reasons for declining treatment were the need to shave the head (50%), the device’s visibility (37.5%), and potential interaction with other treatments (25%).

The study by Zeng and colleagues highlights that patients with brain metastases (*n* = 23) deciding between stereotactic brain surgery and stereotactic brain surgery plus whole-brain radiotherapy considered all treatment outcomes related to QoL and disease control important, as ranked on a self-developed questionnaire. QoL was ranked the highest, followed directly by maintaining functional independence and survival. Items ranked as most important were generally associated with QoL (ie cognition, side-effects). Factors associated with disease control (ie preventing existing and new metastases from growing) were ranked lower by most patients.^32^ However, the concept of QoL in this study was not defined for the patients and was, therefore, open to the participants’ interpretation and items were likely interrelated (eg maintaining functional independence *due to* disease control). Dorman and colleagues^35^ conducted semi-structured interviews to explore the treatment priorities of patients with brain metastases originating from nonsmall cell lung cancer (*n* = 9). Patients’ preferences could be grouped together as follows: (1) achieving the best possible QoL for as long as possible, (2) prolonging life regardless of QoL, and (3) living life to the fullest followed by a rapid decline, avoiding a prolonged dying phase. In addition, they mentioned several important factors for QoL, namely family, mobility/movement, body image or self-image, cognitive function, freedom, normality, and ability to work. Patients expressed fear of losing their dignity, becoming dependent, being a burden, being pitied, or being in pain.^35^

### Information needs

Regarding the decision-making process, PwBT expressed various information needs. The study of Reinert and colleagues^40^ used a self-developed questionnaire to evaluate information needs. They found that 59.9% of a mixed sample of PwBT (*n* = 172) wished to be fully informed. Other patients did not wish to know everything but preferred a comprehensive explanation of diagnosis and treatment (18.6%) or did not want to be informed at all (21.5%).^40^ In a study by Finocchiaro and colleagues,^43^ 68.4% of 18 PwBT (not further specified) reported in semi-structured interviews that they wanted to be informed about their condition, while 15.5% would rather not be informed but have the information provided to a relative. Similarly, patients with brain metastases (*n* = 124) in the study by Papadakos and colleagues^34^ reported a marked preference for information on symptom management, the location of the tumor(s), and medical procedures and follow-up. Patients prioritized medical and physical information, followed by practical and emotional domains, while information on social and spiritual matters were rated as least important.^34^ Patients with nonmalignant intracranial life-threatening lesions (*n* = 25) in the study by Rozmovits and colleagues^39^ expressed in semi-structured interviews that they wanted more detailed and elaborate information than had been provided, and many felt this was important for them to reach a decision about surgery and to cope following surgery.^39^ Besides medical information about treatment options, an overview of the actual surgical procedure, and surgical risks, patients also wanted to know about the neurosurgeon’s background, reputation, and experience.^39^ Furthermore, these patients indicated that they needed more information regarding recovery, both short-term and long-term, and felt the information provided on this topic was insufficient for assessing whether their postoperative symptoms were concerning and required urgent attention.^39^

In the study by Boele and colleagues on high-grade glioma patients (*n* = 15), patients at various stages in their disease trajectory explained in semi-structured interviews that they wanted to know whether treatment was the best or the only option.^22^ They were desperate to seize any opportunity for better outcomes and were often eager to discuss alternative or experimental treatments. Additionally, information regarding treatment procedures and side effects was considered essential to enable patients to organize their life around it. Patients with recurrent glioma (*n* = 15) in the study by Sørensen von Essen and colleagues^11^ expressed in semi-structured interviews that the way the neurosurgeon balanced the information and words used to describe the tumor or surgical procedure affected patients’ risk assessment and, ultimately, the treatment decision. Another group of high-grade glioma patients (*n* = 19), in the study of Halkett and colleagues, expressed in semi-structured interviews that the uncertainty about prognosis made decision-making difficult; however, they did value the opportunity to gather information and ask questions.^23^ In the study by Cavers and colleagues,^26^ glioma patients at various stages in the disease trajectory expressed in serial interviews conducted over 1 year, that they were conflicted between wanting information and not feeling prepared to receive difficult news. In other studies, both patients with recurrent high-grade glioma tumors (*n* = 15) through semi-structured interviews, and a mixed group of PwBT (*n* = 172) through self-developed questionnaires, stressed that receiving adequate information delivered in an understandable manner was critical for decision-making.^11,40^ Importantly, the preferences regarding the type and preferred amount of information differed between patients, as was emphasized in 3 studies, highlighting the importance of individually tailored information.^11,23,33^ This involves providing patients with details about their diagnosis, treatment options, recovery process, and potential risks in a way that resonates with their personal needs and preferences. For example, some patients may prefer detailed technical explanations, while others may prefer simpler, more visual information.^11^

### Practical and emotional support needs

Four studies on glioma patients emphasized the potential diminished medical decision-making abilities of patients.^11,23,24,26^ Their ability to fully understand the information provided^11,23^ may be affected by their cognitive impairments due to the disease and prior treatments,^23^ or their emotional response to the diagnosis.^24,26^ As noted in the serial interview study by Cavers and colleagues,^26^ the ability to absorb information was often affected, especially in the early phase where shock and a cognitive “shutdown” inhibited information absorption and retention. Especially recurrent high-grade glioma patients with aphasia or other cognitive impairments demonstrate a higher dependency on family caregivers to speak on their behalf or assist them in other ways to make a decision, as reported by both patients (*n* = 15) and family caregivers (*n* = 14) in semi-structured interviews.^11^ According to patients, family caregivers play a crucial role in decision-making by providing emotional and practical support, such as gathering information or even taking responsibility for decision-making, as emphasized in 2 qualitative studies on patients with high-grade gliomas.^11,23^ Similarly, Succop Jr. et al. (2023) found that glioma patients (*n* = 20) relied on loved ones and support groups when deciding whether to pursue treatment.^27^

Furthermore, patients highlighted a few other important aspects regarding decision-making, such as the need to maintain a sense of control.^22^ In the study by Sterckx and colleagues, high-grade glioma patients (*n*=17) reported in semi-structured interviews feelings of disregard when they felt excluded from decision-making by their family caregivers or HCP or when they felt family caregivers were preventing them from becoming well-informed.^25^ The interview study by Boele and colleagues^22^ highlights opportunities to augment patients’ sense of control, by supporting them to plan life around treatments, explore experimental treatment options, and reserve the option to stop or pause treatment.

Another important decisional support need of patients was maintaining hope.^11,26,33^ In the study by Sze and colleagues^33^ on patients with brain metastases (*n* = 20) and the study by Halkett and colleagues^23^ on high-grade glioma patients (*n* = 19), hope was defined by patients as more than hope for a cure or survival, but also hope to improve symptom management, reach acceptance, or sustain QoL. In the study by Sørensen von Essen and colleagues,^11^ recurrent high-grade glioma patients (*n* = 15) and family caregivers (*n* = 14) emphasized in semi-structured interviews that HCPs should never take away a patient’s hope. As reported by Boele and colleagues^22^ patients with glioma (*n* = 15) felt that treatment risks were overemphasized relative to benefits. Cavers and colleagues^26^ concluded from serial interviews that glioma patients (*n* = 26) strategically sought positive information to maintain hope, especially early in the disease trajectory. However, patients in these interviews perceived HCPs as lacking sensitivity and emotional support in this regard, focusing predominantly on medical procedures.^26^

In the semi-structured interviews conducted with patients with nonmalignant but life-threatening brain tumors (*n* = 25) by Rozmovits and colleagues,^39^ patients also shared the need for compassion from their neurosurgeon. In a similar fashion, the interview studies by Sørensen von Essen and colleagues^11^ and Boele and colleagues^22^ conducted semi-structured interviews and highlighted the importance of a trustful relationship with the HCP(s) for high-grade glioma patients. Patients expressed that having trust in the HCP could directly influence the likelihood of choosing surgery. In a study of a mixed PwBT sample (*n* = 42) by Goebel and Mehdorn,^42^ patients completed a questionnaire on communication preferences regarding bad news, identifying 3 key preferences: being told in person, receiving the doctor’s full attention, and maintaining eye contact. These preferences all reflect patients’ need for emotional connection.

### Decision aids and interventions

From 2018 to 2024, 6 studies have examined decision aids and interventions to improve decision-making for PwBT (Table 2). Two studies focused on enhancing information provision to patients. Hertzsprung and colleagues^45^ investigated the impact of stereoscopic visualization, a type of patient-specific 3D imaging, during surgical consent consultations in a mixed sample of PwBT (*n* = 27). Using a self-developed questionnaire, they found no significant improvement in patients’ decision-making experience or objective knowledge regarding surgical procedures. In contrast, van de Belt and colleagues^31^ found through semi-structured interviews that glioma patients (*n* = 11) reported predominantly positive experiences with the use of 3D tumor models in consultations, particularly for enhancing their understanding of the relationship between the tumor and brain structures, as well as the surgical procedure and associated risks.

Four studies assessed decision aids or interventions aimed at improving SDM by fostering patient participation in the decision-making process. Shepherd and colleagues^29^ explored the consultation planning, recording, and summarizing approach (CPRS) in high-grade glioma patients (*n* = 12) at an early stage in their treatment trajectory. The CPRS method involves preparing for the consultation with a coach by listing personal questions, concerns, and key information to address during the upcoming medical consultation. The consultation is then audio-recorded and later summarized in plain language by the coach for the patient. Through repeated semi-structured interviewing, the researchers found that it helped patients be involved and share meaningful information. However, patients did not feel they had a meaningful choice in their treatment, and it was improbable they would disregard their doctor’s advice. Patients found it reassuring and emotionally supportive to understand the rationale for the proposed treatment plan. Another study by Bergers and colleagues^44^ involving patients with various brain tumors (*n* = 42) provided a content-analysis of a digital person-centered care tool. The tool included 5 open-ended questions for patients to answer regarding important activities now and in the future, important people in their lives, healthcare-related concerns, key information their HCPs should know about them, and their expectations of treatment. Answers were shared with their HCP through the electronic health record. The analysis of what patients filled out in the tool underscored the importance of maintaining normal everyday activities, such as sports or social activities, and addressing patients’ concerns during decision-making regarding disease impact on daily life.^44^ It also provided insight into what patients wanted HCPs to know, such as treatment goals or communication preferences.^44^ Leu and colleagues^46^ studied an SDM intervention for patients with high-grade glioma, low-grade glioma, and brain metastases. The SDM intervention included a training for HCPs and the implementation of a decision grid, a visual tool used in medical consultations that lists options along with their main benefits and risks. However, the authors reported no significant increase in SDM, as measured with the collaborate questionnaire, possibly due to the questionnaire’s lack of sensitivity.^46^ Lastly, Sørensen von Essen and colleagues conducted an alpha-test to assess the acceptability and usability of a patient decision aid designed to support SDM for high-grade glioma patients (*n* = 11), their family caregivers (*n* = 9), and HCPs (*n* = 11).^30^ The decision aid was based on a generic SDM template developed by the Center for SDM in Denmark.^48^ It is a paper-based decision aid, including a preparation sheet for the consultation and a folder outlining the 5 steps of the decision-making process to be used during the consultation. Inside the folder are individual options cards, summarizing the benefits and disadvantages of each option in brief sentences accompanied by pictograms. Additionally, a card with patient narratives is included to help patients reflect on their values and preferences related to the decision. Participants expressed positive feedback on the decision aid’s clinical applicability, with patients and family caregivers reporting a high perceived preparedness for decision-making, reflected in a mean score of 80.83 (on a scale from 0 to 100).

## Discussion

This scoping review shows that: (1) although there is variability in the decisional roles that PwBT wish to adopt in the decision-making process, most prefer a collaborative or an active role in decision-making; (2) patients value treatment outcomes concerning QoL, functional independence, and survival when making treatment decisions, with individual preferences shaped by potential risks and the impact on daily living; (3) adequate, understandable information is crucial for decision-making, and calls for individually tailored amount and type of information; (4) PwBT need support from their HCPs and family caregivers in the decision-making process to maintain hope, to establish a trustful relationship with their HCP, and to cope with diminished medical decision-making abilities; and (5) studies that explored decision aids and interventions to improve decision-making for PwBT either focused on information provision (eg through videos, 3D models), or SDM by focusing on involving patients in decision-making, with mixed effects on patient participation and satisfaction.

The preferences for decisional roles of PwBT are slightly more active in comparison to a large meta-analysis from 2010 that administered the CPS to various cancer populations at different stages in their disease trajectory. This analysis, including 3491 cancer patients across 6 clinical studies,^49^ reported that 25% preferred a passive role in decision-making in comparison to 14%–29% in PwBT, 49% a collaborative role in comparison to 28%–46% in PwBT, and 26% an active role in comparison to 32%–58% in PwBT. The preference for a collaborative or active role may be explained by the severity of the disease and the impact of treatment decisions on daily life.^36^ Moreover, patients anticipate a more active role in the later stages of the disease when options for life prolongation become more limited, and QoL takes precedence.^36^ However, the selected role seems to also depend on how patients interpret the different roles. As found in qualitative studies, PwBT interpreted collaborative or active involvement as becoming well-informed, being able to voice what matters to them, and choosing between provided alternatives.^22,36,38,39^ Additionally, the characteristics of the decision at hand may also influence the roles that patients select. Neuro-oncological decisions often involve high-impact treatment choices that carry uncertainty regarding treatment outcomes for QoL, including risks and benefits of surgical intervention, and, for glioma patients, lack curative options and require patient commitment to follow through with treatment.^1^ According to a recent review, these decision characteristics make SDM appropriate in this context.^50^ What might hamper active patient participation is that PwBT and their HCPs, as reported in qualitative studies, feel as if they have little choice due to the limited available treatment options.^22,33^ In certain cases, palliative care might provide a meaningful alternative, even though it is not a tumor-directed treatment.^51^

PwBT stressed QoL as an important treatment outcome.^32^ One study discerned 3 groups of patient preferences: (1) achieving the best possible QoL for as long as possible, (2) prolonging life regardless of QoL, and (3) living life to the fullest and avoiding a prolonged dying phase.^35^ Acknowledging individual patient preferences for treatment outcomes, including their goals regarding QoL and survival, is essential for improving the quality of treatment decisions by aligning treatment with these preferences and goals.^52–54^ Yet, patient advocates expressed that care was not always aligned with patient preferences regarding aggressiveness of treatment, personal goals or milestones in life.^24^ Although the importance of QoL is widely acknowledged, it is still mostly regarded as a secondary endpoint to survival in clinical trials and is not yet fully integrated into clinical decision-making.^55,56^ A study amongst 396 oncologists reported that, despite considering QoL findings of randomized controlled trials useful, they were infrequently used to guide clinical decisions with patients.^57^ This was due to a lack of time (67%), insufficient understanding of QoL outcomes (57%) and concerns about the generalizability of results (68%). The limited knowledge about the impact of treatment on daily life complicates the discussion of trade-offs between QoL and survival in clinical practice.^56,58^ Nevertheless, research addressing QoL and functional independence outcomes following treatment, which may support these discussions, is expanding,^55,57,59^ including studies on specific treatment outcomes regarding daily life, such as return to work^5^ or resuming social roles.^60^ More research is needed to understand how these group findings will translate to predictions of individual outcomes.^61^ The trade-off discussed should be guided by patient preferences regarding treatment outcomes elicited by the HCP.^55^

Regarding information preferences, adequate information is crucial for decision-making. However, the amount and type of information PwBT prefer differs. The need for tailored information is incorporated into 26 of 40 existing SDM models, as described in a review of key elements of SDM models, highlighting its importance.^9^ However, there is no consensus about the extent and manner to inquire about patients’ information needs regarding treatment, treatment outcomes, risks, and prognosis.^62^ Therefore, standardized frameworks and communication practices need to be developed to guide HCPs in eliciting and responding to patients’ preferences. An example is the decision-aid developed by Sørensen von Essen and colleagues, where patients can indicate the amount of information they wish to receive about treatment options.^30^ The importance of providing patients with clear information is demonstrated by an RCT, in which a video on life-sustaining measures (such as cardiopulmonary resuscitation) in the active dying phase, led to significantly more patients with recurrent glioma choosing comfort care over life-prolonging or basic care.^15^

Studies emphasize the need for support by HCPs and family caregivers for PwBT during decision-making, especially regarding their potentially diminished decision-making capacity. PwBT have been found, already in the early disease trajectory, to struggle to understand medical information and weigh alternatives.^7,63^ However, HCPs tend to overestimate decision-making abilities of PwBT early in the disease trajectory,^4^ whereas with recurrent disease, HCPs are more aware of diminished decision-making capabilities and tailor the amount of information if necessary to prevent confusion.^64^ Cognitive testing might prove useful to flag patients at risk for diminished decision-making abilities to support HCPs in estimating patients’ capabilities.^4,6^ Regarding emotional support, maintaining hope and establishing a trustful relationship with their HCP are recurrent topics for patients. In clinical practice, setting realistic goals (eg taking a trip with good symptom control, spending time with family by limiting hospital appointments) has been suggested as a strategy to maintain hope,^65^ as well as the use of decision-making support tools for HCPs. For example, the serious illness conversation guide comprises a list of questions to support HCPs in having conversations with patients about their goals, prognosis, and information preferences.^66^

Several limitations should be noted concerning this scoping review. We performed our search in PubMed, which covers only a portion of the published medical literature. Moreover, the terminology used for decisional preferences and needs varied considerably, often overlapping with terms commonly found in titles and abstracts on unrelated topics. Therefore, despite thoughtful effort, we may have failed to include all relevant studies. Only one study referred to the ODSF,^11,12^ a framework of decisional needs and preferences, which could help standardize results for future studies on this topic. Furthermore, there was large heterogeneity between studies with respect to methodologies, patient samples, and inherently different treatment tradeoffs for patients with different brain tumor diagnoses. Many studies used a qualitative design involving patients at different stages of the disease trajectory. This approach provides valuable in-depth insights yet complicated result integration due to the lack of standardized outcomes, making comparison and synthesis difficult. Additionally, the reviewed studies often provided results based on combined groups of patients with different diagnoses, family caregivers, and/or HCPs together, whereas their needs and preferences may be vastly different.^43^ This highlights the need for further research to disentangle the distinct needs of these groups. Moreover, selection bias may have been introduced in decision-making studies, as participants tend to be more engaged and able to participate actively. Lastly, no studies addressed the self-efficacy of PwBT or family caregivers, a decisional need described in the ODSF.^12,13^

To conclude, SDM could help address the needs and preferences of PwBT, summarized in this review, within the decision-making process. Moreover, QoL and functional independence are crucial yet underexplored factors in decision-making for PwBT. Further research is needed to better integrate individual patient outcome preferences into SDM and to evaluate practical tools that support informed and value-based decisions.

## Supplementary material

Supplementary material is available online at *Neuro-Oncology Practice* (https://academic.oup.com/nop/).

npaf056_suppl_Supplementary_Appendix_S1

npaf056_suppl_Supplementary_Appendix_S2
