# Supplementary material for: A scoping review: Understanding brain tumor patients’ decisional needs and preferences
Source: Neurooncol Pract. 2025 Jun 17;12(6):933–51. doi: 10.1093/nop/npaf056 (PMC12741842; doi:10.1093/nop/npaf056)
Supplement: npaf056_suppl_Supplementary_Appendix_S1 [file npaf056_suppl_supplementary_appendix_s1.docx]

# Appendix 1 Search terms PubMed

| Database | Search strategy |
| --- | --- |
| PubMed (PubMed incl. MEDLINE): 1659 records | (decision*[Title/Abstract])  AND  ("brain neoplasms"[Title/Abstract]  OR brain tum*[Title/Abstract]  OR "meningioma"[Title/Abstract]  OR glio*[Title/Abstract]  OR "neuro-oncology"[Title/Abstract]  OR "brain cancer"[Title/Abstract]  OR brain metastas*[Title/Abstract])  AND  ("goals"[Title/Abstract]  OR "wants"[Title/Abstract]  OR "needs"[Title/Abstract]  OR "preference"[Title/Abstract]  OR involve*[Title/Abstract]  OR engage*[Title/Abstract]  OR "participation"[Title/Abstract]  OR "wishes"[Title/Abstract]  OR "priorities"[Title/Abstract]  OR "expectation"[Title/Abstract]  OR concern*[Title/Abstract]  OR value*[Title/Abstract]  OR "matters"[Title/Abstract]  OR "support"[Title/Abstract]  OR "knowledge"[Title/Abstract]  OR patient decision*[Title/Abstract]  OR "patient centered care"[Title/Abstract]  OR "patient-centered care"[Title/Abstract]  OR "quality of life"[Title/Abstract]  OR "quality-of-life"[Title/Abstract]  OR "decision aid"[Title/Abstract])  NOT  ("pediatric"[Title/Abstract]  OR "child"[Title/Abstract]  OR "adolescent"[Title/Abstract]) |
